# Resting-state EEG networks characterized by intramodular and global hyperconnectivity in depressive sample

**DOI:** 10.1192/j.eurpsy.2022.557

**Published:** 2022-09-01

**Authors:** A. Komarova, A. Kiselnikov, M. Yurlova, E. Slovenko, I. Tan, D. Mitiureva, P. Kabanova, E. Terlichenko, V. Zubko, E. Shcherbakova

**Affiliations:** Lomonosov Moscow State University, Psychology, Moscow, Russian Federation

**Keywords:** Depression, Resting-state EEG, Connectivity

## Abstract

**Introduction:**

Depression is characterized by a pattern of specific changes in the network organization of brain functioning.

**Objectives:**

We researched a graph structure specificity in a depressive student sample by analyzing resting-state EEG. All possible combinations of graph metrics, frequency bands, and sensors/sources levels of networks were examined.

**Methods:**

We recorded resting-state EEG in fourteen participants with high Beck Depression Inventory score (24.4 ± 9.7; 20.4 ± 1.5 y.o.; 14 females; 1 left-handed) and fourteen participants with a low score (6.8 ± 3.7; 21.3 ± 2.0 y.o.; 8 females; 1 left-handed). We applied weighted phase-lag index (wPLI) to construct functional networks at sensors and sources levels and computed characteristic path length (CPL), clustering coefficient (CC), index of modularity (Q), small-world index (SWI) in 4-8, 8-13, 13-30, and 4-30 Hz frequency bands. We used Mann-Whitney U-test (p < 0.05) to investigate between-group differences in the graph metrics.

**Results:**

The depressive sample was characterized by increased CC and Q in the 4-30 Hz band networks and decreased CPL in the beta-band network (sensors-level for CPL and CC, and sources-level for Q).

**Conclusions:**

Elevated CC and Q may relate to an increase of intramodular connectivity, and CPL reduction reflects the global connectivity increasing. We hypothesize that intramodular hyperconnectivity could explain the rise of global functional connectivity in participants with depressive symptoms. *Funding: This research has been supported by the Interdisciplinary Scientific and Educational School of Lomonosov Moscow State University ‘Brain, Cognitive Systems, Artificial Intelligence’.*

**Disclosure:**

No significant relationships.

